# Using participatory action research to empower district hospital staff to deliver quality-assured essential surgery to rural populations in Malawi, Zambia, and Tanzania

**DOI:** 10.3389/fpubh.2023.1186307

**Published:** 2023-09-14

**Authors:** Chiara Pittalis, Grace Drury, Gerald Mwapasa, Eric Borgstein, Mweene Cheelo, John Kachimba, Adinan Juma, Kondo Chilonga, Niamh Cahill, Ruairi Brugha, Chris Lavy, Jakub Gajewski

**Affiliations:** ^1^School of Population Health, Institute of Global Surgery, RCSI University of Medicine and Health Sciences, Dublin, Ireland; ^2^Nuffield Department of Orthopedics, Rheumatology and Musculoskeletal Sciences, University of Oxford, Oxford, United Kingdom; ^3^Deparment of Surgery, College of Medicine, Kamuzu University of Health Sciences (former University of Malawi), Blantyre, Malawi; ^4^Surgical Society of Zambia, Lusaka, Zambia; ^5^East Central and Southern Africa Health Community, Arusha, Tanzania; ^6^Department of Surgery, Kilimanjaro Christian Medical Centre, Moshi, Tanzania; ^7^School of Medicine, University of South Carolina, Greenville, SC, United States

**Keywords:** participatory action research, essential surgery, anesthesia, obstetrics, trauma, nursing, sub-Saharan Africa, engaged research

## Abstract

**Background:**

In 2017 the SURG-Africa project set out to institute a surgical, obstetric, trauma and anesthesia (SOTA) care capacity-building intervention focused on non-specialist providers at district hospitals in Zambia, Malawi and Tanzania. The aim was to scale up quality-assured SOTA care for rural populations. This paper reports the process of developing the intervention and our experience of initial implementation, using a participatory approach.

**Methods:**

Participatory Action Research workshops were held in the 3 countries in July–October 2017 and in October 2018–July 2019, involving representatives of key local stakeholder groups: district hospital (DH) surgical teams and administrators, referral hospital SOTA specialists, professional associations and local authorities. Through semi-structured discussions, qualitative data were collected on participants’ perceptions and experiences of barriers to the provision of SOTA care at district level, and on the training and supervision needs of district surgical teams. Data were compared for themes across countries and across surgical team cadres.

**Results:**

All groups reported a lack of in-service training to develop essential skills to manage common SOTA cases; use and care of equipment; essential anesthesia care including resuscitation skills; and infection prevention and control. Very few district surgical teams had access to supervision. SOTA providers at DHs reported a demand for more feedback on referrals. Participants prioritized training needs that could be addressed through regular in-service training and supervision visits from referral hospital specialists to DHs. These data were used by participants in an action-planning cycle to develop site-specific training plans for each research site.

**Conclusion:**

The inclusive, participatory approach to stakeholder involvement in SOTA system strengthening employed by this study supported the design of a locally relevant and contextualized intervention. This study provides lessons on how to rebalance power dynamics in Global Surgery, through giving a voice to district surgical teams.

## 1. Introduction

An estimated 95% of people in low and middle-income countries (LMICs) lack access to safe surgery, with most unmet need among rural populations, especially in Sub-Saharan Africa (SSA) (1). To meet this need the World Health Assembly recommended expanding the capacity of district-level hospitals (DHs) (2). As the first point of contact for common conditions amenable to surgery, DHs can play a key role in enhancing surgical service coverage and financial protection for rural populations, particularly marginalized and hard-to-reach groups, thus advancing the goal of universal health coverage.

However, workforce shortages and increased pressures on health systems are limiting factors in SSA. These have led to increased professional differentiation at district hospitals, with task-sharing/task shifting of specialist duties to non-specialists (e.g., non-physician clinicians – NPCs and generalist medical officers – GMOs) (3, 4). The cost of surgical training of non-specialists is considerably less than the cost to train specialist surgeons (5, 6). For common, selected surgical procedures, they can achieve similar (effective and efficient) outcomes to SOTA specialists (7), if trained and supervised (4, 8). Yet, non-specialist surgical providers at DHs often work in isolation with few opportunities for professional development and support (9, 10), leading to concerns over the quality and safety of their work once deployed (11).

To address this gap, in 2017 the SURG-Africa project set out to institute a surgical, obstetric, trauma and anesthesia (SOTA) care system strengthening intervention in Zambia, Malawi and Tanzania, focused on non-specialist workforce training, supervision and mentoring (protocol published elsewhere (12)). The aim was to design, implement and evaluate in-service training models to scale up quality-assured SOTA care, appropriate to district hospitals.

In this paper we report the process of developing the in-service training model and our experience of initial implementation, using an inclusive and participatory approach.

### 1.1. Strategies to maximize benefits

Both the Lancet Commission on Global Surgery (11) and national policies (13, 14) state that the ability of non-specialist providers to consult with specialists is critical to the success of task-sharing/shifting models of SOTA care delivery. Quality control systems need to be in place, especially for the management of complicated or unusual surgical or anesthetic procedures. In theory, in the study countries, this should be done through outreach programs, during which SOTA specialists from central and provincial levels periodically visit district facilities to manage the backlog of advanced cases and provide supervision. Our intervention aimed to introduce a more efficient and innovative approach to outreach, designed to go beyond simple supervision visits by specialists to undertake cases at district hospitals; and instead to promote the transfer of knowledge and skills from the specialists to the non-specialist providers, to gradually build local capacity to handle a wider range of SOTA cases and improve referral practices. This was achieved through prioritizing in-service training in visits from specialists.

Additionally, to increase efficiency and speedy and informed decision-making, a managed consultation network was established to ensure regular communication among specialist supervisors and non-specialist district providers in-between visits. Consultations supported collaborative decision-making (whether or not to operate locally, correct case management or referral preparation); and enabled better coordination across care levels in case of patient referral (12). Several strategies were adopted to ensure the relevance, acceptability and sustainable benefits of the intervention as described below.

#### 1.1.1. Development of the training approach

Firstly, the development of the training model was informed by key principles from adult learning and behavioral theories (15). Training sessions included a theoretical and practical aspect, since interactive sessions, which allow participants to practice and hone skills, are more effective at influencing change in professional practice and health care outcomes than purely didactic sessions (16). Training was organized in small groups to allow interaction, experimentation and critical reflection, given the compelling evidence that adults change practice by active learning rather than being taught (17–19).

In line with social influence theories, which assert that individuals’ beliefs and behaviors are influenced by persons in their social network and society at large (20), the intervention was delivered by respected peer leaders, i.e., specialist SOTA providers from central and general hospitals, who oversaw the referral networks of the participating DHs. The use of local specialists, rather than international experts (21), was an explicit strategy to maximize the long-term sustainability of the intervention.

Critically, local government rates were applied for the specialists delivering the program of visits, to facilitate implementation by the ministry of health (MoH) after the end of the project. Also, all supervisors were trained in pedagogy, feedback, supportive supervision, communication and adult learning techniques. This empowered them to be agents of change within their networks, facilitating transmission of the model to their referral hospital colleagues, necessary for supervision in a scaled up program.

#### 1.1.2. Promoting engagement and local ownership

Adult learning theory states that adults perform better on learning tasks that are meaningful and which fall within their domain of interest (22). Therefore, adult learners should be involved in planning curricular directions and should be encouraged to diagnose their own learning needs and objectives, to increase the relevance of the training content and stimulate learners’ motivation to engage (22).

This principle resonated with our desire to make the training program as relevant as possible to the needs of the different cadres of district level SOTA providers taking part in the intervention. It was also important for us to ensure alignment of the intervention with the needs and priorities of the wider SOTA care system stakeholders in the study countries, including local authorities, professional associations and hospital administrators.

Recent discourses on the colonial legacies of global health have highlighted that health interventions that are donor driven and not locally owned risk inadvertently supporting the delivery of healthcare in a way that undermines local care system sustainability and heightens dependence on external help (23, 24). Sustainable system strengthening interventions necessitate feasible and realistic approaches, informed by local MoH and stakeholders’ views of and proposed solutions to their health problems (23); and require them to take ownership and leadership of the interventions (25). This requires their active engagement throughout the research process.

Therefore, SURG-Africa adopted a participatory action research approach as an overall conceptual umbrella to the design, implementation and evaluation of the in-service training and supervision intervention and associated research. Participatory action research (PAR) is a form of collaborative inquiry enabling those involved in the problem under study to be part of the research process; and the research is conducted *with* people rather than *on* them (26, 27). The aim is to study and foster positive change in a particular group, organization or team (28) by democratizing knowledge production and fostering their empowerment (29).

Participatory research approaches have been widely and effectively applied to address public health priorities (30), health systems strengthening and to improve health workforce performance (31). However, to our knowledge, application of this methodological approach in the field of Global Surgery research is new and can provide valuable lessons for strengthening SOTA care for neglected populations in SSA.

## 2. Methods

We held iterative PAR workshops in each country at baseline (design), midpoint (implementation) and endline (evaluation) stages of the training intervention. This paper focuses only on the baseline and midpoint PAR workshops, and explores how stakeholders and researchers used the PAR findings to co-develop and strengthen the intervention design, specific to each country.

### 2.1. Workshop participants

In the design phase, 2-day PAR workshops were held in Zambia in July 2017, in Malawi in August 2017 and Tanzania in October 2017. Follow up PAR workshops (1 day) were held in October 2018–July 2019. These were attended by a total of 119 stakeholders at baseline and 123 at midline across Zambia, Malawi and Tanzania. Attendees included surgical teams as well as hospital administrators from the 31 intervention district hospitals and the main referral central and provincial hospitals. In this paper the term ‘surgical team’ refers to the following professionals: surgical, trauma and anesthesia care providers, theater nurses, obstetricians and gynecologists, as applicable to each hospital level.

Attendees were selected by hospital managers overseeing each participating facility on the request of the research team. They needed to be either surgically active or a representative from a district or central level hospital who could help identify potential implementation, governance, and communication challenges for the intervention, so that these could be addressed locally at the earliest opportunity. Representatives of national surgical, anesthetic, nursing and obstetric professional bodies, and of ministries of health, were also invited to contribute to the discussions. A breakdown of workshop participants is provided in Supplementary File 1.

### 2.2. Targets

The purpose of the baseline PAR workshops in each country was threefold:

- To gain a better understanding of the local context in which the intervention was to be delivered and how this may affect implementation – i.e. policy and operating environment, local surgical care systems’ factors, SOTA conditions commonly presenting, extent of existing district hospital SOTA training and supervision.
- To explore the needs (especially training needs) and challenges of district surgical teams in the delivery of care, and proposed solutions, based on their own lived experience and perceptions.
- To co-design the intervention: presenting the proposed model and eliciting reflection, discussion and joint agreement on how this could be improved and delivered.

The midpoint workshops aimed to jointly review and reflect on the intervention implementation and to identify areas needing further modification, in an inclusive manner. In doing so, they also served to validate the findings of the baseline workshops and the overall in-service training model.

Sets of questions for the baseline and midpoint workshops were developed by the research team around these themes; the full list is reported in Supplementary File 2.

### 2.3. Delivery of the workshops

**Figure 1:**
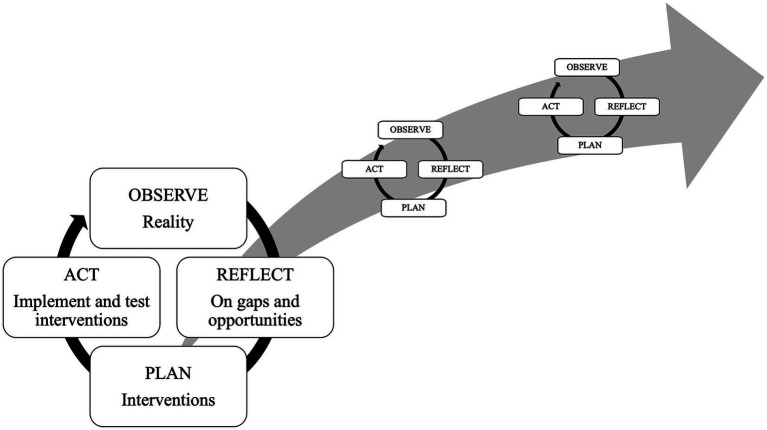
The PAR cycle.

PAR research follows a cyclical process of observation, reflection, action, evaluation/critical analysis and modification (Figure 1), with each cycle yielding new insights or improvements. PAR begins with “small” cycles that address comparatively minor questions or problems before participants move on to more complex or consequential issues (28).

In this study, the baseline workshops represented the first set of ‘cycles’. Researchers and stakeholders joined forces to develop knowledge to inform practice and solve concrete problems - in our case how to expand SOTA care delivery for rural communities through district hospital capacity building. This feature of PAR ensures that the actions of those involved are better informed, if not changed, through the research process (27, 30). Hence, PAR is transformative rather than merely informative (27).

Through a series of group work sessions and plenary discussions, structured around a set of carefully predetermined questions (Supplementary File 2), workshop participants were led through a process of collaboratively:

- Gaining situational awareness of the DH surgical system and common challenges and priorities.
- Exploring the current provision of training, supervision, and mentoring available to DH surgical teams.
- Identifying gaps and opportunities for training, supervision, and mentoring that could be addressed by the intervention.

This was then used to inform the refinement of the intervention and agree on an action plan for the supervisory visits.

The workshops firstly involved group work with DH mentees on their own, i.e., those who would receive supervisory visits, pairing the representatives from 2 DHs. This was followed by group discussions involving DH mentees, central/provincial hospital mentors and other stakeholders. The PAR workshops were iterative in that mentees had the opportunity to discuss questions on day 1 and then these were followed up on day 2, when mentors and other stakeholders joined the discussions.

This sequence was to allow DH surgical teams the time and opportunity to gradually build up their confidence as a group, to enable them to share their views with more senior specialists from higher-level hospitals, and with national level stakeholders, in a safe space. This encouraged open communication and the overcoming of hierarchical and sociocultural barriers. We also used cadre-specific group discussions to elicit experiences and views common across DH cadres such as anesthesia providers, surgical care providers and theater nurses. Each round of group work was followed by a plenary session (as in Table 1) to validate group observations through collective discussions and consensus.

**Table 1:** Overview of the PAR workshop.

Overview of the PAR workshop		
Day 1 - District hospital teams (i.e., mentees) only	Day 2 - All participants	Outputs
Introductions, overview of the proposed intervention model and PAR process (setting expectations and clarifying limitations) – *Mentee groups by pairs of district hospitals:* Discussion 1: Snapshot of the surgical system in the country Discussion 2: What supervision and mentoring is happening in hospitals, and explore possibilities – Plenary – Discussion 3: Training (reflection on existing training, and needs and possibilities) Discussion 4: Detailed planning for the SURG-Africa intervention (developed timetable for 2-day visit) – Plenary	Introductions, the role of supervision, mentoring and training to scale up safe surgery, and recap of previous day’s proceedings – Presentations on DH training, supervision and mentoring needs from the perspective of referral hospitals – Presentations on the role of the supervisory teams – *Mentees-mentors and other stakeholder groups split by cadre (surgery/trauma/obstetrics, anesthesia, theater nursing):* Discussion 1: Supervision and mentoring needs and opportunities for DHs Discussion 2: Training needs and opportunities for DHs – Final discussion (all together) on intervention design	Draft action plan/timetable for the visits tailored to each intervention DH Recommendations for the intervention design Identified key contacts at district and referral hospitals, for logistical planning and implementation

The midpoint PAR workshops followed a similar structure but discussions were condensed into 1 day. The focus was on reviewing progress in the intervention to-date, sharing experiences, lessons learned and identifying any areas for improvement. Action planning and recommendations for the coming 6 months of SURG-Africa visits were made.

### 2.4. Data collection and analysis

The workshops were run at a central location in each country, facilitated by a team of 5–6 (international and local) researchers and key representatives of the stakeholders groups. Data sources collected included: the participants’ written notes of their group discussions, the visit plan templates, the facilitators’ notes and transcriptions of verbal presentations given by each discussion group.

No individual identifiable information was collected and only aggregated data (from the breakout sessions or plenary discussions) were gathered and analyzed. To avoid deductive disclosure, the breakout groups were identified using numerical codes (e.g., group 1, 2, 3 etc.). Two researchers collated the written qualitative data from the three workshops into MS Word and spreadsheet documents. A thematic content analysis was conducted in the software QSR Nvivo 11, a qualitative research management tool. The research targets (as above) and group work questions (in Supplementary File 2) were used to guide the analysis, this was then enriched with new emerging nodes during the coding process.

## 3. Results

### 3.1. Local context and operating environment

An important issue that emerged from the baseline PAR workshop discussions was the skills, availability and management of the different staff cadres in the sample DHs across the three countries. Most hospitals reported uneven skills level across staff cadres and reliance on few skilled personnel for SOTA services. While there was an adequate pool of general nurses available in DHs in the three countries, it was reported that theater nurses were commonly rotated within hospitals and provided cover whenever necessary rather than being permanently assigned to theater duties. This hindered continuity of service and nurses’ ability to acquire and retain essential theater skills.

Stakeholders stated that the type of SOTA procedures done in individual hospitals depended on the availability of skilled staff, but they were in insufficient numbers to deal with the volume of work. Intermittent availability of surgical supplies and breakdown in critical equipment were also common across the three countries.

All these factors affected the quantity and quality of surgeries performed at the district level. They also increased the frequency of referrals of cases to higher care levels, contributing to congestion at central level facilities and higher costs for patients.

Stakeholders confirmed that, at the time, no national guidelines or set standards were in place to guide the work of the district surgical team, and they felt that the development of such material would be extremely helpful in improving the quality of surgical care. International guidelines on quality of care, such as the WHO surgical safety checklist, were known but usually not followed.

### 3.2. Experience of previous in-service training, mentoring, and supervision

All workshop groups across the three countries reported a lack of in-service training available to develop essential skills such as pre, intra and post-operative care, and the surgical skills to manage common surgical, obstetric and trauma conditions seen at DHs. They also reported limited or no training opportunities in the use and care of surgical and anesthesia equipment, essential anesthesia care and infection prevention control. The groups reported a lack of access to mentoring, and lack of feedback on surgical cases referred to higher-level facilities.

In regards to supervision, some of the workshop participants reported that on-the-job support was offered by occasional visiting doctors, often from NGO programs (e.g., AMREF flying doctors or ONSE project in Central Malawi). However, priority in these visits was usually given to clearing the surgical lists rather than on the sharing of knowledge with district hospital staff. Also, these programs were often irregular, due to overstretched government resources or intermittent donor funding. Despite the limited scope of such initiatives and their irregularity, when supervisory visits did take place in some hospitals, participants stated that the visits were appreciated as this enabled preparation of cases and some (albeit limited) learning. A reported drawback was that there was often not much feedback and no communication on the patient’s progress after these visits.

### 3.3. Action planning and co-design of the intervention

Through an action-planning cycle the workshop participants identified and prioritized the training needs that could be addressed by the project through regular in-service training, mentoring and supervision visits over an intervention period of 24 months. The participatory action process led to critical changes and adjustments to the intervention design, as follows.

#### 3.3.1. Training content

##### 3.3.1.1. Original proposal

The Lancet Commission on Global Surgery proposed a core set of high volume, cost-effective essential procedures which, being less technically complex, could be delivered at DH level (11). Hence, the training was to be focused on these essential general surgical and obstetric procedures, and trauma management and stabilization, with the aim of increasing the range of SOTA services offered at district level. In addition, the training was to cover a range of non-technical skills such as management skills, professionalism, patient-centeredness, data collection and use of surgical information systems.

##### 3.3.1.2. Post-PAR intervention design

The workshop groups recommended that, given the varying skills level of district surgical teams, the training content should include new essential SOTA procedures. But it was also important to provide refresher training on core SOTA skills, as well as addressing other knowledge gaps. Examples of priority training areas identified in the workshops are presented in Table 2.

**Table 2:** Examples of reported district training needs and priorities.

Reported training needs and priorities		
Surgical, trauma, obstetric providers	Anesthesia providers	Nurses working in theater
Basic surgical skills Advanced Trauma Life Support training (in Tanzania) Emergency cases (surgical and obstetric) Resuscitation Investigations and diagnostics, including referral decision-making Ultrasound training	Recognition and management of the severely injured patient Pre-, intra- and post-operative care (including preparation of patient, spinal and general anesthesia, monitoring) Record keeping and data Fluid management Using anesthesia equipment	Skills to deal with surgical emergencies Basic handling of surgical instruments Familiarization with common surgical procedures presenting at district level Essential skills on scrubbing, gowning, gloves and use of drapes Post-op care

In terms of non-technical skills, the groups emphasized that rather than focusing on hospital management teams or other staff, in the first instance the training should start with the surgical teams. They identified the need to encourage more teamwork within the district hospital teams, as well as across care levels, since district and higher level hospitals tended to work in isolation, with little communication and coordination. The basics of infection prevention and control were to be reiterated, including promoting the use of the surgical safety checklist as standard practice. Attention was also needed on ways to promote accountability and reduce reluctance to embrace new practices.

#### 3.3.2. Training visits design

##### 3.3.2.1. Original proposal

To develop an in-service training guide and curriculum that would be followed as standard by all of the participating district hospitals for each training visit.

##### 3.3.2.2. Post-PAR intervention design

It was decided that the training visits were to be planned according to the training needs identified by each intervention hospital as most urgent. This implied changing the intervention approach from a top-down, standard training model to an individualized and needs-based one. Each DH team and their mentors developed a typical visit itinerary and range of activities/components and discussed logistics that worked best for their specific hospital needs.

#### 3.3.3. Timing of training visits

##### 3.3.3.1. Original proposal

The DHs would have periodic (3–6 monthly) field visits from mentors.

##### 3.3.3.2. Post-PAR intervention design

Negotiations during the workshop determined the duration (2 days) and the frequency (every 3 months in Zambia and Tanzania, every 2 months in Malawi) for the visits. These discussions took into account the SOTA specialists’ availability, visit costs and feasibility to subsequently determine the best visit time frames.

#### 3.3.4. Mentees for training

##### 3.3.4.1. Original proposal

The training was aimed at a broad range of DH staff involved in the provision of and support to surgery at DHs, including: NPCs and doctors who undertake surgeries, anesthetic staff, post-operative ward nurses, administrators responsible for supply chain management, maintenance, transport, medical records, management information systems.

##### 3.3.4.2. Post-PAR intervention design

To maximize the limited time available during the visits and to ensure meaningful (and impactful) engagement, the workshop groups recommended that the training approach be more targeted, focusing on clinical staff (SOTA providers and theater nurses) in the first instance. This was based on an understanding of the challenges of existing team dynamics and training gaps gathered during the PAR workshop, and the recognition of the need to offset the influence of staff rotation and turnover on retention of district surgical teams’ knowledge.

#### 3.3.5. Mentors for training

##### 3.3.5.1. Original proposal

The mentors would be specialist surgeons working at referral hospitals, supported by one or more anesthesiology and obstetric specialists.

##### 3.3.5.2. Post-PAR intervention design

The workshop groups determined which cadres and how many mentors should be in the visiting specialist team, with country-specific team composition. Instead of 1–2 mentors in each team, the teams were expanded to 3–4 persons to include a theater nurse alongside the surgeon and anesthetist, and often additionally included either an orthopedic surgeon (in Tanzania) or an obstetrician as needed.

In Malawi, theater nurse training was identified as most urgent, but there was limited capacity locally. Hence, a decision was made to invite theater nursing specialists from Zambia to deliver training of trainers in Malawi to build up local capacity to deliver the intervention.

#### 3.3.6. Training curriculum

##### 3.3.6.1. Original proposal

The training curriculum was to be organized around a range of standalone modules adapted from: (i) a Primary Trauma Course already in use in several countries in the region (32); and (ii) the Essential Surgical Training curriculum being run by the College of Surgeons of East, Central and Southern Africa (COSECSA) as a district hospital training program (33).

The mentors would use the Primary Trauma Care courses and Essential Surgical Training curriculum for each visit. During those visits, the mentors would cover the knowledge, skills, and attitudes in surgical skills and competencies as agreed by national stakeholders and the essential general surgery and obstetrical surgery, and trauma management and stabilization.

##### 3.3.6.2. Post-PAR intervention design

Workshop groups discussed stand-alone courses such as Primary Trauma Care, but it was clear that other knowledge gaps were to be addressed and not all district teams were at the same level or perceived the same needs. The choice to proceed with an individualized, needs-driven approach to training required more flexibility in the curriculum than that originally planned. The groups also emphasized their preference for practical, rather than didactic training. Elements of primary trauma care training were incorporated into the visits rather than centralized courses.

The workshop groups agreed to prioritize the mentees’ needs rather than the mentors’ preferences, through targeted, personalized mentoring that addressed the mentees’ weaknesses. To this end, mentors and mentees were to agree learning goals together to work toward, and jointly agree the ‘terms of reference’ of the working relationship, so that expectations were clear and realistic.

The workshop groups stated that another critical consideration for the success of the intervention was the need to focus: (1) on supportive supervision, rather than top-down supervision, which was typical in some medical disciplines. This required developing honest and clear communication between mentors and mentees; and (2) on the district surgical team as a whole, rather than individual cadres, since SOTA care delivery is a “team” effort.

### 3.4. Midpoint PAR proceedings

The midpoint PAR workshops offered an opportunity for stakeholders to reflect on their experience of the intervention to that point and to review progress. The main takeaway message was that overall the (co-designed) intervention model was working well. They observed a number of positive developments such as growing confidence of district surgical teams in the delivery of SOTA care; initial improvements in skills for the selected procedures being practiced with the mentors; increased independent planning of procedures; and reduction in unnecessary referrals thanks to better case management and decision-making practices.

Yet, some challenges persisted, particularly in regards to intermittent availability of supplies and piped water (in Malawi especially). In some cases, the visiting supervisory teams were able to bring with them some supplies to mitigate shortages, but this was not a lasting solution.

Other challenges noted in the implementation of the intervention included: district mentees not being always available during the supervisory visits or not adequately prepared; too few cases booked for the visits to practice; visits postponed or canceled due to scheduling conflicts between mentors and mentees; lack of sufficient care in the post-op recovery areas, including unavailability of monitors; challenges in ensuring that the right person takes responsibility for the theater (e.g., when nursing tasks were task-shifted to the caretaker and cleaner rather than being undertaken by an assigned nurse, with risk of quality/safety being compromised); and the need to streamline consultations over the remote consultation network (in Malawi).

Lessons learned and agreed action points for the future were:

- To foster better communication with District Health Management Teams and district hospital management to address shortages of surgical and anesthesia supplies and equipment, and to encourage better resource planning.
- To facilitate more efficient communication between mentors and mentees in the project’s remote consultation network to streamline requests for advice. To this end, it was recommended for the project to introduce the SBAR (Situation-Background-Assessment-Recommendation) technique. All teams were to be duly trained on SBAR and the protocol was to be shared.
- To promote better visit planning – mentors and mentees recognized the need to work closer together to eliminate causes for visit cancellations/rescheduling. District teams were reminded to ensure adequate bookings and patient preparations in view of the visits.
- To ensure continuity of mentorship (i.e., particular mentors assigned to particular district hospitals) as this was important for relationship building and trust.

## 4. Discussion

The unmet need for SOTA care remains worryingly high in SSA. Our project aimed to address this need by expanding existing service delivery at the district hospital level through a capacity building intervention designed with the input of local stakeholders. The aim of this paper is not to promote one choice of intervention over others, but rather to highlight the value of using an inclusive and participatory approach to the design and implementation of SOTA system-strengthening interventions.

This study used Participatory Action Research to co-design country-specific models of district-level surgical workforce training, supervision and mentoring in Malawi, Zambia and Tanzania. To our knowledge, this is the first use of a PAR framework in the design of a DH in-service training intervention in sub-Saharan Africa.

We intentionally used “local expertise” to bridge practice and research (34), through PAR workshops that brought together health workers from a range of hospital levels, from consultants and specialists working at central hospitals to surgical teams at DHs, to share perspectives. The latter – the mentees who were to receive the supervision – were at the center of the process. Non-clinical decision-makers such as ministry of health representatives were also included in the discussions.

The workshops involved learning by all participants, respecting and valuing diversity in opinions (among different cadres of health providers and different stakeholder groups); supporting interactions among groups that would not normally have opportunity to come together (9) (e.g., district hospital clinicians and referral hospital clinicians); and addressing the importance of local as well as national contexts in health system strengthening interventions.

We sought to mitigate power imbalances by using local facilitators as far as possible, allowing small group discussions that built up over consecutive days; asking participants to summarize their discussions in their own words with their own nominated representatives and asking for representation and contributions from all cadre groups. The workshops involved open discussions and extensive group work (facilitated by researchers) to encourage participants’ reflective practice, i.e., cycles of learning, reflection and action about their own experiences. This built self-awareness and promoted creativity in finding solutions to shared problems; and ultimately empowered participants to be actors for change.

From the project perspective, the PAR workshops formed and strengthened relationships between future mentors and mentees and helped to set expectations about the purpose and scope of the intervention. They identified which parts of the district surgical system might be directly addressed by the intervention, and areas which could not be addressed. For example, the project could not provide new health workers, equipment or infrastructure. This collaborative work also developed improved guidance on what procedures should be done at the district level and which should be referred, which is still a topic under debate in many SSA countries (35).

From the surgical system perspective, this multi-disciplinary and multi-level dialog, bridging hierarchies based on seniority and location (central-district level), so as to agree solutions, helped to improve rapport and communication among stakeholders on how to build SOTA care capacity in Malawi, Zambia and Tanzania. The face-to-face interactions enhanced appreciation of the roles and challenges each group faced. Among other things, this helped redefine the role of SOTA specialists – not just “as someone to refer cases to” for the technical and clinical skills of their specialty, but also as trainers and mentors. The supportive professional relationships established by the project provide opportunities for collaboration across hospital levels beyond the life of the project.

Overall, the PAR process in our study allowed the shift of power from those who are more powerful, including specialists, national managers and the research team, to the intended beneficiaries of the intervention – the district surgical teams. These teams identified key priorities, set the direction for change, and refined the final design of the intervention to become more relevant and aligned to their needs. The workshops contributed to defining priority skills and competencies for each district hospital institution and for each cadre.

By adopting a bottom-up approach where the intervention was selected and shaped by local stakeholders’ needs and worldviews, rather than imposing top–down solutions, this study enabled the development of a more appropriate and potentially sustainable intervention. It is also a more ethical and equitable approach to health research, than where the interventions are pre-determined and the evaluation process is tightly controlled by the researchers.

Indeed, participatory health research has been commended as a useful tool to overcome what is known as “epistemic injustice” (36). These are situations where certain kinds of ‘knowers’ are seen as less credible than policy-makers and professionals; and their knowledge is not taken seriously into account, leading to single or one-sided views of reality (37). In PAR the research is done with stakeholders, who are regarded as expert-by-experience (30). As shown in this paper, our study and approach offers a practical example of how to address the power imbalances that are key to the design of sustainable health systems interventions; and, as a by-product, to more equitable health research by enabling all stakeholders to participate in the research process (23, 38).

In our study the PAR process was critical to ensuring the relevance, feasibility and acceptability of the intervention, and its chances of success and sustainability. The final results of the intervention evaluation are presented in a separate publication (forthcoming) but, as shown here, without the comprehensive involvement of the relevant stakeholders’ in the co-design process, the intervention would not have fully captured and responded to needs on the ground, with risk of wasted investments. This is particularly important in Sub-Saharan Africa, where public health resources are limited and there is a large unmet need for surgical care.

The use of adult learning theories to inform the intervention, the reliance on local supervisors, and alignment to local government rates for costing the visits, were other important strategies to promote local ownership of the intervention and the chances of its continuation beyond the life of the project.

There are potential limitations in the PAR approach, including the possibility of researcher bias, as the authors were involved in the design of the workshops, and were present at the workshops. However, we sought to minimize this through using local senior SOTA specialists as facilitators, with workshop participants given maximum opportunity in plenary sessions to give verbal summaries and group discussions. To minimize subjectivity, two researchers independently undertook thematic analysis of the qualitative data generated at the PAR workshops.

Despite these limitations, our findings suggest that participatory action research approaches are useful in SOTA systems research, especially when needing to compensate for power hierarchies that can lead to the beneficiaries’ needs being neglected, and instead ensuring the design of a user-focused (i.e., stakeholders) intervention. This study has generated many methodological and practical lessons that can be of value to others wishing to pursue stakeholders’ involvement in their research and contributes to the global discourse on more equitable global surgery research.

Our PAR findings have been disseminated to national decision makers to support them in making policy decisions and presented these to members of professional bodies, such as national surgical and anesthesia societies, and representatives from Ministries of Health at subsequent workshops in Zambia, Malawi, and Tanzania.

## Data availability statement

The datasets presented in this article are not readily available because to protect the confidentiality and privacy of the participants. Requests to access the datasets should be directed to chiarapittalis@rcsi.ie.

## Ethics statement

Ethical approval was granted by the Research Ethics Committee of the Royal College of Surgeons in Ireland (approval no. REC1417), the College of Medicine Research Ethics Committee in Malawi (approval no. P.05/17/2179), the University of Zambia Biomedical Research Ethics Committee (approval no. 005–05-17), the Kilimanjaro Christian Medical College Research Ethics and Review Committee (approval no. CRERC 2026) and the National Institute for Medical Research in Tanzania (approval no. NIMR/HQ/R.8a/Vol. IX/2600).

## Author contributions

CL, GD, JG, RB, EB, JK, and KC conceived the study. GM, MC, and AJ supported the organization and running of the workshops, CL, GD, JG, EB, JK, and KC facilitated the workshops. CP, GD, GM, MC, and AJ collected the data. CP and GD analyzed the data and wrote the first draft of the manuscript, with support from NC. All the authors proofread the manuscript and approved the final version.

## Funding

This study was undertaken as part of the SURG-Africa project funded by the European Commission Horizon 2020 Framework Program (Grant number: 733391).

## Conflict of interest

The authors declare that the research was conducted in the absence of any commercial or financial relationships that could be construed as a potential conflict of interest.

## Publisher’s note

All claims expressed in this article are solely those of the authors and do not necessarily represent those of their affiliated organizations, or those of the publisher, the editors and the reviewers. Any product that may be evaluated in this article, or claim that may be made by its manufacturer, is not guaranteed or endorsed by the publisher.
