# Supplementary material for: Using participatory action research to empower district hospital staff to deliver quality-assured essential surgery to rural populations in Malawi, Zambia, and Tanzania
Source: Front Public Health. 2023 Sep 14;11:1186307. doi: 10.3389/fpubh.2023.1186307 (PMC10536269; doi:10.3389/fpubh.2023.1186307)
Supplement: Supplementary file 1 [file Data_Sheet_1.DOCX]

Supplementary File 1 – Workshop participants

Using participatory action research to empower district hospital staff to deliver quality-assured essential surgery to rural populations in Malawi, Zambia and Tanzania

Chiara Pittalis^*^, Grace Drury, Gerald Mwapasa, Eric Borgstein, Mweene Cheelo, John Kachimba, Adinan Juma, Kondo Chilonga, Niamh Cahill, Ruairi Brugha, Chris Lavy, Jakub Gajewski

*** Correspondence:** Chiara Pittalis: [chiarapittalis@rcsi.ie](mailto:chiarapittalis@rcsi.ie)

The SURG-Africa intervention targeted surgical teams (including SOTA providers and theater nurses) from 31 district hospitals (DH) in selected research sites in two regions in Northern Tanzania, Kilimanjaro Region and Arusha Region (12 DHs), the Southern Region of Malawi (9 DHs), and the Southern Province in Zambia (10 DHs).

At baseline, representatives from all intervention DHs took part in the PAR workshop in Malawi. In Zambia and Tanzania the number of DHs represented at the first PAR workshop (6 and 15 DHs respectively) differed from the final number of DHs in the intervention group (10 and 12 DHs respectively) as the selection process was still ongoing at the time of the baseline PAR and situation analysis. Representatives from all the 31 intervention DHs took part in the midline PAR workshops.

| *Participants:* | **Malawi** | **Tanzania** | **Zambia** |
| --- | --- | --- | --- |
| **Baseline PAR** | August 2017 | October 2017 | July 2017 |
| District hospitals representatives | 18 (from 9 intervention DHs) | 40 (from 15 DHs) | 12 (from 6 DHs) |
| SOTA and nursing specialists from central/provincial hospitals | 10 (from Queen Elizabeth, Zomba and Kamuzu central hospitals) | 9 (from Kilimanjaro Christian Medical Centre, Arusha Lutheran Medical Centre, Mt. Meru Regional Referral Hospital, Muhimbili National Hospital) | 12 (from Lusaka University Teaching Hospital, Livingstone Central Hospital, and Mazabuka, Choma, Monze and Maamba general hospitals |
| Professional associations representatives | *** | 5 | 6 |
| Ministry of Health / provincial authorities representatives | 1 | 4 | 2 |
| Total stakeholders: | 29 | 58 | 32 |
| **Midpoint PAR** | March 2019 | July 2019 | October 2018 |
| District hospital representatives | 18 (from 9 DHs) | 29 (from 12 DHs) | 18 (from 10 DHs) |
| SOTA and nursing specialists from central/provincial hospitals | 22 (from Queen Elizabeth, Zomba and Kamuzu central hospitals) | 14 (from Kilimanjaro Christian Medical Centre, Arusha Lutheran Medical Centre, Mt. Meru Regional Referral Hospital) | 16 (from Lusaka University Teaching Hospital, Livingstone Central Hospital) |
| Professional associations representatives | *** | 3 | 3 |
| Total stakeholders: | 40 | 46 | 37 |
| **Given the low number of SOTA specialists in Malawi, many of the specialists invited to the workshops were also representatives of their respective professional associations. They are considered once in this table to avoid double counting.* | | | |
