# Supplementary material for: Using participatory action research to empower district hospital staff to deliver quality-assured essential surgery to rural populations in Malawi, Zambia, and Tanzania
Source: Front Public Health. 2023 Sep 14;11:1186307. doi: 10.3389/fpubh.2023.1186307 (PMC10536269; doi:10.3389/fpubh.2023.1186307)
Supplement: Supplementary file 2 [file Data_Sheet_2.DOCX]

Supplementary File 2 – Guide for PAR group discussions

Using participatory action research to empower district hospital staff to deliver quality-assured essential surgery to rural populations in Malawi, Zambia and Tanzania

Chiara Pittalis^*^, Grace Drury, Gerald Mwapasa, Eric Borgstein, Mweene Cheelo, John Kachimba, Adinan Juma, Kondo Chilonga, Niamh Cahill, Ruairi Brugha, Chris Lavy, Jakub Gajewski

*** Correspondence:** Chiara Pittalis: [chiarapittalis@rcsi.ie](mailto:chiarapittalis@rcsi.ie)


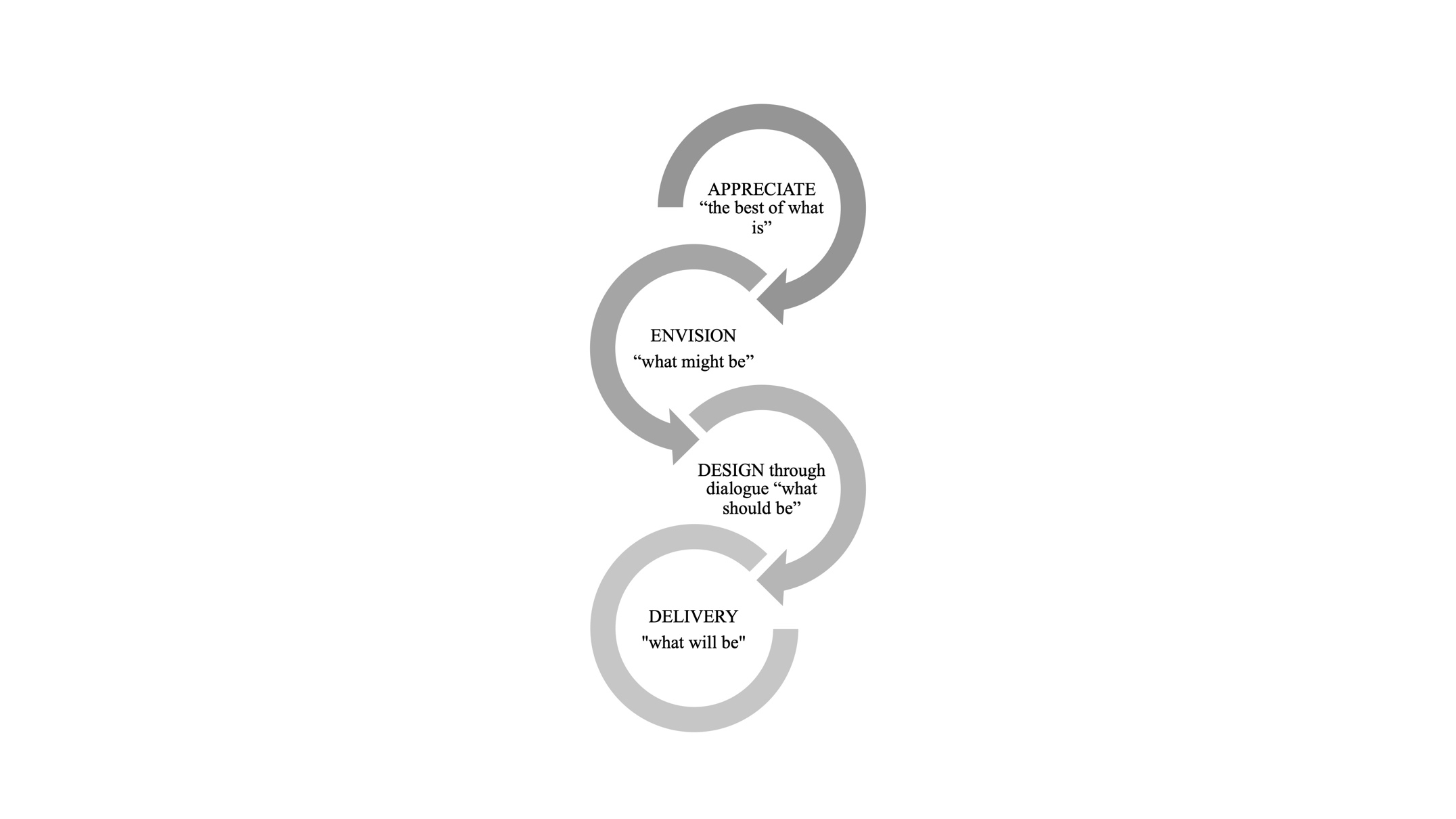
Within the PAR framework reported in Figure 1 in the manuscript, we developed specific sub-questions using the principles of Appreciative Inquiry (first articulated by Cooperrider and Whitney in 2005(1)), which are commonly used for planning, implementing and evaluating change processes in healthcare (2).

**Figure S1. Principles of Appreciative Inquiry.**

Questions and discussion topics used by the project to facilitate the group work consisted of the following:

**BASELINE WORKSHOPS**

1. OBSERVE AND REFLECT at hospital level – increasing situational awareness of district hospital surgical systems
   1. Changes
      1. The most significant changes in your hospital recently (past 6 months) and the impact of these changes on the surgical system
   2. Challenges
      1. The hospital’s main challenges
   3. Priorities
      1. The hospital’s key short/medium/long-term priorities
2. OBSERVE AND REFLECT on experiences of supervision, mentoring and training for district hospital surgical teams - at individual, cadre and team level.
   1. What “is”?  (Reflection on history/experience)
      1. Describing current experiences of supervision and mentoring in the DH surgical team
      2. Analyzing what is (and is not) working well
      3. Exploring different cadre perspectives and needs (multi-disciplinary approach)
      4. Assessing short/long-term training needs
      5. Identifying gaps in skills/knowledge/attitude for each cadre
3. EXPLORE POSSIBLE ACTIONS - What “could be”?  (Explore possibilities for change)
   1. Prioritizing skills/knowledge/attitudes to build surgical capacity/quality in DH surgical teams
   2. Considering options for intervention format and components - how supervision, mentoring and training could be delivered to DH surgical team, including identifying opportunities for on-the-job training and centrally coordinated training events
   3. Defining quality (to maximize potential impact) – the features of good training, supervision and mentoring for DH surgical teams
4. PLAN SPECIFIC ACTIONS - What “will be”? – Planning for change
   1. Reaching consensus on the learning objectives, surgical oversight team composition, and components of the regular SURG-Africa supervision, mentoring and training visits (specific for each cadre)
   2. Context-specific planning - create an itinerary and training plan for a 2-day supervision/mentoring visit for each hospital
   3. Consideration of implementation factors – (logistic, administrative, stakeholders, communication, equipment, coordination, transport)

**MIDPOINT WORKSHOPS**

1. OBSERVE AND REFLECT on experiences of supervision, mentoring and training for district hospital surgical teams
   1. What is your experience of mentoring during the visits?
   2. What is your experience of mentoring in between visits?
   3. What new skills have you learned from the visits?
   4. Have you had the opportunity to practice these?
   5. Have you received meaningful feedback on your performance?
   6. Have there been learning points from the remote consultation network?
2. EXPLORE POSSIBLE ACTIONS - What “could be”?  (Explore possibilities for change)
   1. What skills do you want to prioritize learning in the next few visits?
   2. Have there been any missed opportunities for learning, during the visits? How could learning opportunities be maximized further?
   3. Are there some operations you’ve done while the visiting team were there, that you wouldn’t have done otherwise?
   4. Are there some operations you’ve done since the visits started, as a result of increased skill/confidence levels?
   5. Have there been adequate supplies for the visits? What could be improved?
   6. Have there been implementation issues? How could these be addressed?
   7. Have the visits met your expectations? If yes/no - why / why not?
3. PLAN SPECIFIC ACTIONS - What “will be”? – Planning for change
   1. How could planning/preparation for visits be improved?
   2. How could follow/up to the visits be improved?
   3. Has teaching been well planned and delivered? If yes, what went well? If not, what could be improved?

**References**

1. Cooperrider D, Whitney D. Appreciative inquiry: a positive revolution in change. 1st ed. San Francisco: Berrett-Koehler Publishers Inc.; 2005.

2. Trajkovski S, Schmied V, Vickers M, Jackson D. Using appreciative inquiry to transform health care. Contemp Nurse. 2013;45(1):95–100.
